# Disambiguation of patent inventors and assignees using high-resolution geolocation data

**DOI:** 10.1038/sdata.2017.64

**Published:** 2017-05-16

**Authors:** Greg Morrison, Massimo Riccaboni, Fabio Pammolli

**Affiliations:** 1 Department of Physics, The University of Houston, Houston, Texas, USA; 2 IMT Institute for Advanced Studies, Lucca, Italy; 3 Department of Managerial Economics, Strategy and Innovation, K.U. Leuven, Leuven, Belgium; 4 Politecnico di Milano, Milan, Italy

**Keywords:** Technology, Economics

## Abstract

Patent data represent a significant source of information on innovation, knowledge production, and the evolution of technology through networks of citations, co-invention and co-assignment. A major obstacle to extracting useful information from this data is the problem of name disambiguation: linking alternate spellings of individuals or institutions to a single identifier to uniquely determine the parties involved in knowledge production and diffusion. In this paper, we describe a new algorithm that uses high-resolution geolocation to disambiguate both inventors and assignees on about 8.5 million patents found in the European Patent Office (EPO), under the Patent Cooperation Treaty (PCT), and in the US Patent and Trademark Office (USPTO). We show this disambiguation is consistent with a number of ground-truth benchmarks of both assignees and inventors, significantly outperforming the use of undisambiguated names to identify unique entities. A significant benefit of this work is the high quality assignee disambiguation with coverage across the world coupled with an inventor disambiguation (that is competitive with other state of the art approaches) in multiple patent offices.

## Background & Summary

In many contexts, technological progress and innovation is essential to national or regional economic growth and output^1^. One way of measuring innovation is the production of patents, which represent a technological advancement produced by individuals (generally, these are inventors on the patents) working at research institutions (generally, these are the assignees on the patent). The analysis of bibliographic databases has provided techniques for evaluating information spillovers^2–4^, mobility between regions^5–7^, interregional and international collaborations^6,8–11^, and the emergence of new technologies^12^ in the context of patent and publication activities. Among many others, these studies have provided an in-depth picture of the dynamics of regional and institutional talents and quantify the success of various inter-organizational and inter-regional collaboration, of great use to policy makers on the institutional, regional, and international level.

A major problem in the use of patent data (or any bibliometric database, such as for scholarly publications^13,14^) is the disambiguation of authors and institutions. There are a wide range of alternate spellings of a person’s or institution’s name, where, for example, ‘The National Institutes of Health’ and ‘NIH’ may refer to the same institution. Typos and misspellings of names are also common in bibliographic data (e.g., ‘National Institute of Health,’ missing an ‘s’ in the second word). The goal of disambiguation is to link all of these alternate spellings of institutional or individual names *without* incorrectly linking similar names referring to distinct entities. This is a difficult task, as there are millions of names to disambiguate (making pairwise comparisons of the full dataset computationally expensive) and an evaluation of how likely two names on patents are to be the same entity is not known a priori and often relies on machine learning techniques^13,15–18^. This disambiguation problem has been effectively approached in recent years in the context of patent data using Baysian methods^15^, Markov Chain Monte Carlo approaches^19,20^, structural equivalence or other network similarity properties^17,21^, and supervised machine learning^22^, with a significant effort continuing at the USPTO^18^ with a focus on inventor disambiguation using an efficient hierarchical clustering approach^19,20^. Each of these approaches has a differing coverage (generally focused on inventors in the USPTO), and a single approach that effectively disambiguates both assignees and inventors in multiple national patent offices is of great value in disambiguating the names of individuals and institutions globally.

**Figure 1:**
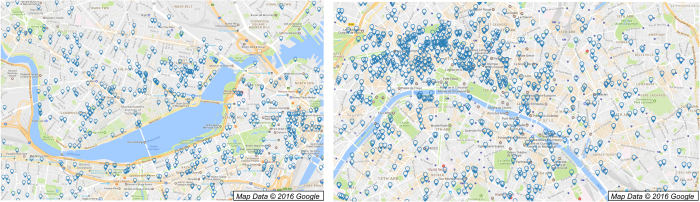
High-resolution geolocations of assignees in the Boston (left) and Paris (right) regions. All high-resolution geolocations in Boston are included (~1.1 K within 10 km of the city center), but due to the density of assignees in Paris, only lat/longs that are seen on at least three patents are shown (~5.2 K within 10 km of the city center). Clusters of geolocations are observed near the Massachusetts Institute of Technology, the Massachusetts General Hospital, Boston University, and downtown Boston.

In this paper, we describe a straightforward but accurate approach to the disambiguation problem using high precision geolocation of assignee and inventor addresses, depicted in Fig. 1 for the Boston and Paris areas. Two inventors (or two assignees) that provide exactly the same high-resolution address and *also* have ‘similar’ names are very likely to refer to the same entity. Thus, knowing that two entities have exactly the same address allows a great deal of flexibility in name matching, and we design two simple string matching approaches to link similar names that share a high-resolution geolocation. Inventors and assignees that have addresses with low resolution, which are extremely common in the US Patent and Trademark Office (USPTO) data, are linked to exact name matches nearby, greatly increasing the coverage of the disambiguation. Additional linking can be accomplished by searching for similar names that share other characteristics in common (such as co-inventors or co-citations). We show that this approach provides a complete disambiguation of inventors and assignees on 8.47 million patents, and that the precision and recall of the resulting disambiguation is superior to or competitive with other well known disambiguation methods.

Our algorithm is one of many approaches to the problem of disambiguation of inventors and/or assignees in patent databases, and differs in many ways from previous methods. Our approach combines a flexible but simplistic name matching algorithm at high resolution geolocations with a stricter name matching at low resolution geolocations applied to both assignee and inventor names, but other previous works use more complex Bayesian or supervised machine learning algorithms. In Table 1, we highlight some of the primary differences between our approach and previous methods. Li *et al.*^15^ used a Bayesian algorithm with an iterative blocking scheme applied only to USPTO inventors, which may outperform our approach in more difficult-to-disambiguate cases. Pezzoni *et al*.^17^ performed a disambiguation of EPO inventors matching similar names that share additional characteristics in common. Ventura^22^ used a large and verified database of pre-disambiguated inventors in the OptoElectronics field to design a supervised learning technique that performs extremely accurately on that subset of the data. The OECD provides the HAN database^23^ that corrects for a variety of alternate spellings and legal distinctions of assignees for patents in the USPTO, EPO, and PCT. Each of these algorithms has been shown to be accurate and useful in certain contexts, but none provide a broad and unified approach to the disambiguation of assignee and inventor names in multiple patent offices.

Our results are a significant improvement over existing disambiguation techniques in a number of ways. This is the first simultaneous disambiguation of names in the EPO, PCT, and USPTO, providing a bridge between two existing but often non-overlapping streams of research focused on European^4,8,10^ and US^12,15,24,25^ patents. This combined disambiguation is of particular use in minimizing bias due to European or American institutions tending to apply for patents in their own domestic offices (the so-called home bias), and ensuring fair coverage of research hubs on both sides of the Atlantic. A disambiguation of both the assignee and inventor names is an aspect lacking in most freely available disambiguations of patent databases. This is of particular importance for understanding the mobility of inventors between institutions (whether in the same region or in different countries), as well as identifying the institutional research hubs that are major players in each country and region. Finally, we emphasize that our disambiguation also incorporates high-resolution geolocations of all inventor and assignee, providing geospatial information that can go beyond traditional regional or national aggregations based on (often arbitrary) administrative boundaries^26^. We believe the breadth and level of detail in the database produced by this work will be of great value to researchers, and will make the data freely available for noncommercial use.

## Methods

### Patent data sources

In this paper, we combine a number of distinct databases covering different patent offices: the US Patent and Trademark Office (USPTO), the European Patent Office (EPO), and patents filed under the Patent Cooperation Treaty (PCT). The USPTO and EPO generally provide protection of intellectual property within the US and European Union (EU) respectively, while the PCT provides a method of international protection for a patent filed domestically. In general, US firms tend to be most represented in the USPTO, European entities in the EPO, and other nations (particularly developing nations) in the PCT database.

Information about USPTO patents is extracted from Harvard’s Dataverse project^15^ from the work of Ref. 15 (covering patents granted between 1975 and 2010), while information about EPO and PCT patents is extracted combining the Organization for Economic Cooperation and Development’s (OECD) RegPat^27^ and Citation^28^ databases (the 2014 version, covering patents applied for between 1977 and 2011). We also incorporate the OECD’s Triadic Patent Families^29^ database in this work, which identifies patents that cover the same technology filed in the USPTO, EPO, and Japanese Patent Offices (JPO). This combination of the USPTO, EPO, and PCT databases provides the application year for each patent, the names and addresses of all inventors and assignees, citations between patents (including those filed in different offices), and patents that are found in the same triadic family. Note there may be no legal requirements or error checking on these addresses. Inventors are free to use their home or office addresses, and assignees can use a PO Box if desired, leading to great heterogeneity in the address fields.

Our method will be compared with two other freely available methods of name cleaning and disambiguation that exist in the literature: for inventor disambiguation we will compare our results to the output of Ref. 15, and for assignee disambiguation we will compare our results to the name cleaning provided by the OECD Harmonized Applicant Name^23^ (HAN) database. This paper will focus heavily on direct collaborations of assignees and inventors, direct citations, or exact family correspondence, and will not use additional information available in the databases such as the technological classes of each patent. Technological classifications have multiple potential levels of aggregation, ranging from very general topics like ‘Pharmaceuticals’ to specific topics like the full International Patent Classification (IPC) code ‘A61K003/121: Medicinal preparations containing acyclic ketones,’ and it is not clear which classification scheme is best for the disambiguation process. Future work may incorporate this additional information (with patent classifications playing a major role in other disambiguation methods^15^), but the high fidelity of our results with benchmarks suggest the incorporation of additional bibliographic data will provide a relatively moderate improvement.

### Geolocation and disambiguation of assignees and inventors

**Figure 2:**
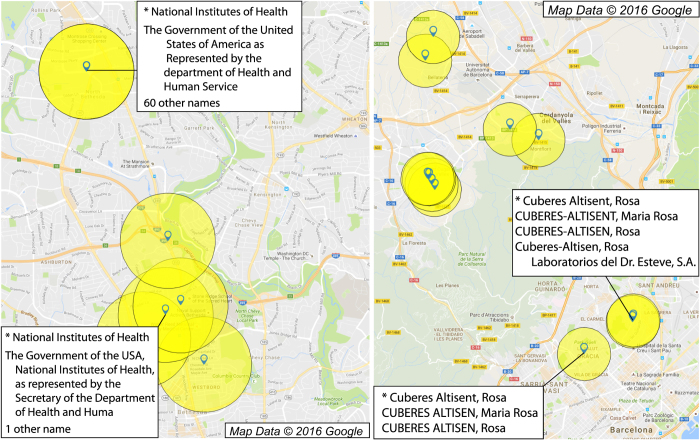
Geolocations of assignee addresses (left) or inventor addresses (right) for two examples. The NIH in Bethesda Maryland, and an inventor in the Barcelona area with many names and addresses. In both cases, there is a great deal of heterogeneity of names at some geolocations, but many of the names are ‘similar’ at precise addresses. There are also exact name matches nearby (within 20 km in this paper), highlighted in bold text.

The fundamental difficulty that must be overcome in name disambiguation is the possibility of alternate or error-ridden spellings of names or addresses in the database. Two examples of names requiring disambiguation are depicted in Fig. 2: on the left are some of the names associated with the National Institutes of Health (NIH) in Bethesda MD (assignee on ~4,900 patents), and some of the names associated with a prolific inventor, Rosa Maria Cuberes-Altisent, in the Barcelona area (inventor on 90 patents). In both cases, the subsets of names shown in the white boxes of Fig. 2 indicate the extreme heterogeneity in some inventor or assignee names, with ~90 unique alternate spellings for the NIH and ~10 unique alternate spellings for Rosa Maria Cuberes-Altisent (removing spacing, punctuation, and capitalization). Disambiguation of these names requires not only matching all of the possible variations in the spelling of the institution or individual, but also *not* matching other names that refer to different entities with similar names. A wide range of methods of varying complexity have been generated to solve this disambiguation problem for authors of publications^13,14,30–33^, patent assignees^16,34,35^, and patent inventor names^15,17^ (with the disambiguation of Li *et al.*^15^ a recent and comprehensive result for the USPTO). In the case of inventor disambiguation, these methods will generally compare pairs of names using the similarity of the text of the names as well as data regarding the assignees, patent citations, patent classes, and geographical information.

**Figure 3:**
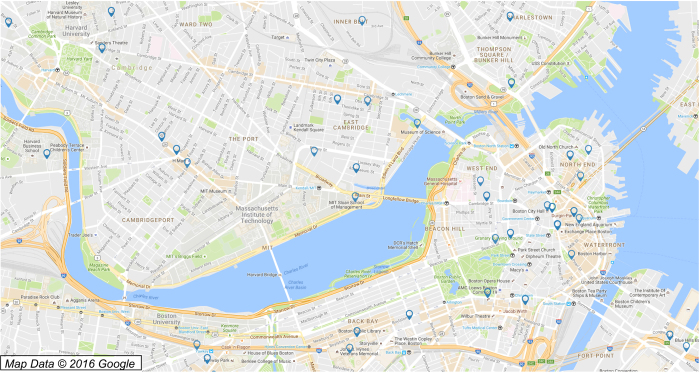
The handful of geolocations of low-resolution addresses in the Boston area. Note that there are ~40 K patents found at these ~180 points, in comparison to the ~15 K patents found at the ~1,100 points in Fig. 1. The loss of specificity in these geolocations would make flexible name matching unreliable at these low resolution geolocations.

The geographical information found in the USPTO typically suffers from low quality addresses, where below 5% of USPTO inventors complete the street field in their address on the patent (city- or zipcode-level information is the highest resolution available). At this resolution, geolocation can be used as one of many rough indicators of the similarity between two names when comparing them for disambiguation (compare the small number of locations in Fig. 3 to the dense coverage of high-resolution locations in Fig. 1). However, patents in the EPO or PCT databases are found to contain higher resolution addresses in a far greater fraction of cases (where a street number, street, city, state, and zip code are often all provided), which can provide much greater specificity when comparing inventors: if two inventors have ‘similar’ names *and* live at exactly the same address, it is far more likely they refer to the same person than if they had ‘similar’ names and lived in the same general area. The same state of affairs exists for assignee names, with the EPO and PCT addresses often having street-level information and the USPTO addresses tending to be of low quality.

**Figure 4:**
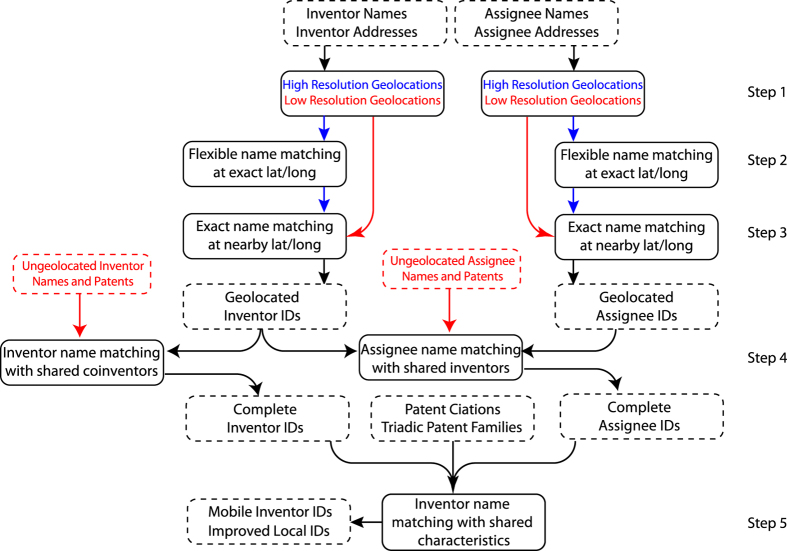
Schematic diagram of the method. Names and addresses are first geolocated (discussed in Sec. 1 of the SI), after which there is a search for ‘similar’ names at exact high resolution lat/longs (the meaning of ‘similar’ is discussed in Sec. 2 of the SI for assignees and Sec. 3 of the SI for inventors). Once similar names are clustered at each lat/long, ‘nearby’ exact name matches are linked (described in Sec. 4 of the SI). Assignees and inventors names that could not be geolocated are linked to disambiguated names with shared coinventorships (described in Sec. 5 of the SI). Finally, similar inventor names for inventors that are not found at the same address but share other characteristics in common before are linked, creating identifiers for mobile inventors (described in Sec. 6 of the SI).

Our strategy for disambiguation will be to leverage these high precision addresses in the EPO and PCT by flexibly matching names that are simultaneously found at exact, high-precision geolocations in any patent office, then to match ‘nearby’ names that are exactly the same. ‘Nearby’ is taken as distance of ≤20 km in this paper, which is a rough upper bound on the mean commute distance in major metropolitan regions in the US^36^. Small variations in this threshold are not expected to significantly alter our results, but a significant decrease or increase in the threshold may cause significantly fewer or more (respectively) links between names. The general idea is sketched in Fig. 2 for specific assignee and inventor names, and a schematic of the methodology is diagrammed in Fig. 4. A detailed summary of each step can be found in the appendix. We first geolocate the assignees and inventor addresses for every patent in the three databases (~4 million unique addresses) using Yahoo’s YQL API^26^, converting the text into likely latitude/longitude (lat/long) pairs and the quality of that geolocation (step 1 in Fig. 4). The quality returned by YQL generally indicates if the geolocation was resolved at the level of a point (e.g., ‘55 Fruit Street, Boston, MA, USA’), line (e.g., ‘Fruit Street, Boston, MA, USA’), zip code (e.g., ‘02114 MA USA’), city (e.g., ‘Boston, MA, USA’), state (e.g., ‘MA, USA’), or country (e.g., ‘USA’). Low quality geolocations are far less informative than high resolution geolocations, with a far greater number and diversity of inventor and assignee names at each low res point than at at a high resolution location (compare Fig. 3 and Fig. 1). In early 2016, Yahoo discontinued the use of the particular API used in this paper, and no longer appears to produce the same quality indicator (see https://developer.yahoo.com/boss/search/#pricing). Other geolocation APIs often provide quality indicators (e.g., the Google Maps API labels geolocations as ‘rooftop’ for the highest resolution, with other indicators for lower resolution). While the results of this paper are strongly dependent on the division between high and low resolution geolocations, we expect our results to be robust to changes of the specific quality indicator used.

For every high-quality geolocation in the data (those better than line-resolution) we attempt to flexibly match similar strings (step 2 in Fig. 4), with specific examples in Fig. 2: assignees with names involving ‘institute’ or ‘health’ at identical lat/longs are likely to be referring to the NIH, and inventors with names like ‘cuberes’ and ‘rosa’ at identical lat/longs are likely referring to Rosa Maria Cuberes-Altisent. The specifics of the matching can be found in Sec. 3 of the SI, but generally speaking we match pairs of assignee names that share either one rare or two common words (in any order, up to one spelling error) and match pairs of inventor names for which the first two words in either name occurs in the other (in any order, up to one spelling error), so long as both names share a high-resolution geolocation. After this first round of disambiguation, which only compares pairs of names found at exactly the same high-resolution geolocations, we search for exact name matches that were geolocated to a lat/long pair of low quality (having a YQL quality indicator on the level of city-resolution) in step 3 of Fig. 4. For example, we link the names at the indicated high-resolution geolocations due to the simultaneous name matchings of ‘National Institutes of Health’ and ‘Cuberes Altisent, Rosa’ occurring in Fig. 2. We also search for inventor name prefixes that clash (where an inventor may leave his or her middle name out on some patents) to prevent incorrect linking of low resolution names. The process is fully described in Sec. 4 of the SI. Steps 2–3 in Fig. 4 are run in parallel to one another, with assignee and inventor disambiguation independent of each other up to this point.

Patents that have assignees that could not be geolocated at the precision of city-level or better are common, particularly in the USPTO data, and it is important to include patents with extremely poor geolocations in the disambiguation as well. Matching assignee names to the disambiguation not straightforward if multiple disambiguated IDs have the same name as the un-geolocated assignee: for example, one must determine whether the assignee ‘General Electric Corporation’ without an address provided (occurring over 18,500 times in the USPTO) is referring the same institution as the ‘General Electric Corporation’ found in New York, Ohio, Massachusetts, or many additional potential geolocations found for that name. We identify the most likely disambiguated ID for linking an unlocated patent as that with a similar name and shared inventor or an inventor in close physical proximity to the geolocated assignee (a detailed description of this method is found in Sec. 5 of the SI).

The results of the disambiguation after step 4 of Fig. 4 produces assignee and inventor IDs that are highly localized, linking names only if their geolocations are within a 20 km radius of one another. For assignees this localized partitioning is reasonable, with different corporate offices, research labs, or subsidiaries of the same company in different cities treated as independent entities (for example, ‘General Electric Corporation’ in Schenectady NY is not the same as ‘General Electric Corporation’ in Cincinnati OH). Inventors are fundamentally different in that they can be mobile, moving between different cities and countries. We therefore perform a final round of linking on the disambiguated inventor IDs in step 5 of Fig. 4 based on characteristics in common that indicate a potential relationship: a shared co-inventor, shared assignee, shared triadic family, or citation. Note that we do not include the technological classes of the patents in our measure of similarity, because they are less personal (working on a research topic is far less informative than collaborating with an individual) and the ‘correct’ level of aggregation is unclear. A link between disambiguated IDs is performed for inventors with identical names and one characteristic or similar names and two characteristics in common, providing a method to identify the movement of unique individuals between different cities.

This procedure produces a disambiguation of ~9.3 M patents in the three patent offices. A total of ~804 K unique geolocated assignee identifiers are found, as well as ~443 K unlocated assignee IDs (due to missing addresses that could not be linked to the disambiguation). We find ~3.8 M unique geolocated inventor IDs that do not move (i.e., are only found in the same 20 km radius), ~425 K mobile inventors (IDs that are linked to geolocations more than 20 km apart), and ~439 K inventor IDs that could not be linked to a location.

The algorithm uses geospatial information throughout, so the unlocated IDs are likely to be error prone and unreliable. Because correction of errors in spelling occurs only in steps 1 and 5 of Fig. 4, it may be important to further distinguish between ‘high-quality’ disambiguated IDs (those involving both geolocation and spelling correction), and ‘low-quality’ disambiguated IDs (those using only geolocations to link nearby identical names). High-quality inventor or assignee IDs can be linked to a high-resolution geolocation, while high-quality inventor IDs also include those linked in step 5 through additional shared characteristics. We find a total of ~290 K high quality assignees and ~1.8 M high quality inventor IDs using this definition, with the best coverage in the EPO and worst coverage in the USPTO (see Table 2). Our method produces a complete disambiguation of all assignees and inventors on ~8.5 M patents, and complete high-quality disambiguations on ~4 M patents worldwide (see Table 3). The disambiguation thus completely geolocates and disambiguates all names on over 90% of the patents in these offices, with nearly 50% of the those patent disambiguations incorporating high resolution addresses and error correction in the names.

## Data records

The results of our algorithm are being made freely available, with the full release found in Data Citation 1. The content and format of each individual data file in the release is described below.

### Patent descriptor file

**Main descriptor file.** File Name: all_disambiguated_patents_withLocal.txt (Data Citation 1)

This is a list of every patent with a disambiguated assignee or inventor in our disambiguation. There are 7 columns and 9.3 M lines, ‘|’ delimited; the columns are:

1. pat: the patent publication number. Each can be linked exactly to either the OECD or USPTO databases for further processing if needed.
2. invs: the inventors on the patent. If multiple inventors are found, they are comma separated.
3. localInvs: the local IDs of the inventors. If no mobile inventor is found, this is identical to the invs column. If multiple inventors are found, they are comma separated.
4. apps: the assignees (applicants) on the patent. If multiple assignees are found, they are comma separated.
5. yr: the application year of the patent.
6. classes: the WIPO industrial fields of the patent. If there were more than one field, they are comma separated. These are integers between 1 and 36, the meaning of which is in the file wipo_class_ID.txt (described in Sec. 3.1.2).
7. wasComplete: a flag to indicate whether too many or too few inventors or assignees were found. A ‘1’ indicates no problem was detected with this patent, a ‘0’ indicates a count difference between disambiguated IDs and names found on the patent. There are 134 K errors of this type in the data.

In the paper, we describe dividing disambiguation into high- and low-quality, based on whether geolocation and name cleaning was performed for that ID. These are indicated by the first two characters in the IDs themselves:

High quality IDs:

- HA: High-resolution assignee (linked in step 1)
- HI: High-resolution inventor (linked in step 1)
- HX: High-resolution cross-linked inventor (linked in step 5)
- HM: High-resolution mobile inventor (linked in step 5)
- LX: Low-resolution cross-linked inventor (linked in step 5)
- LM: Low-resolution mobile inventor (linked in step 5)

Low quality IDs:

- LA: Low-resolution assignee
- HS: High-resolution split inventor
- LI: Low-resolution inventor
- LS: Low-resolution split inventor
- UI: Unlocated inventor
- UX: Unlocated cross-linked inventor
- US: Unlocated split inventor

Each of these two-letter codes are followed by a number:

- For assignees (where the second character is ‘A’), the number indicates that assignee’s rank by total number of patents. In the case of a tie, the rank is alphabetical. For example, HA1 is a high resolution assignee with the most patents in the world, and LA4 is a low resolution assignee with the fourth most patents in the world.
- For local inventor IDs (where the second character is NOT ‘M’ or ‘A’), they indicate the rank of that local ID by total number of patents. In the case of a tie, the rank is alphabetical. For example, HI1 is the most prolific high-resolution inventor in a single 20 km region, while LI2 is the second-most prolific inventor in a single 20 km region.
- For mobile inventor IDs (where the second character is an ‘M’), they indicate the rank among other mobile IDs of that Inventor’s most prolific local ID. For example, LM1 is the mobile inventor with the greatest number of patents in one 20 km region, and all geolocatons for that inventor were low resolution (indicated by the ‘L’). HM2 is the mobile inventor with the second greatest number of patents in one 20 km region, and at least one geolocation was high resolution (indicated by the ‘H’).

### Top-level patent classifications in the patent descriptor file

File Name: wipo_class_ID.txt (Data Citation 1)

This is a list of the link between WIPO fields^37^ and the indicator found in

all_disambiguated_patents_withLocal.txt. There are two columns, 37 lines,‘,’ delimited; the columns are:

1. wipoClass: the top level field
2. ID: the number between 1–36 corresponding to that class.

**Mobile inventor links.** File Name: mobilityLinks.txt (Data Citation 1)

This is a list of local inventor IDs (IDs found within 20 km of one another) to the mobile IDs detected in step 5. 2 columns, 1 M rows,‘,’ delimited; the columns are:

1. localID: local ID of the inventor.
2. mobileID: mobile ID of the inventor.

### Disambiguated IDs to names and locations

**Inventors.** File Name: LinkedInventorNameLocData.txt (Data Citation 1)

This is a list of the patents, geolocations, and names of every inventor. There are 6 columns and 25.3 M lines,‘|’ delimited; the columns are

1. ID: the local ID of the inventor
2. pat: the patent publication ID.
3. name: the raw, undisambiguated name of this inventor on this patent.
4. loc: the geolocation of the inventors address listed on this patent.
5. qual: the yahoo quality of the geolocation.
6. loctype: the division of the geolocation into high and low resolution. If one wishes to work with individual data at a high resolution, do not use ‘low.’

**Assignees.** File Name: LinkedAssigneeNameLocData.txt (Data Citation 1)

This is a list of the patents, geolocations, and names of every inventor. There are 6 columns and 8.2 M lines, ‘|’ delimited; the columns are

1. ID: the ID of the assignee
2. pat: the patent publication ID.
3. name: the raw, undisambiguated name of this assignee on this patent.
4. loc: the geolocation of the assignee’s address listed on this patent.
5. qual: the yahoo quality of the geolocation.
6. loctype: the division of the geolocation into high and low resolution. If one wishes to work with data at a high resolution, do not use ‘low.’

### Patent citations

File Name: Citation_App+Exa.txt (Data Citation 1)

This is a list of patent citations found in the OECD or Dataverse^15^ databases. Non-patent citations (e.g., to journals) are not included. Patent citations can be added by the applicant or the examiner in the office (the latter suggesting it is relevant but the inventor may not have been aware of it). A typical USPTO patent has significantly more outgoing citations than a typical EPO/PCT patent, and depending on the research topic under study, one may need to carefully consider the impact of this difference between US and European offices when looking at citation statistics. 4 columns, 42.5 M lines, ‘|’ delimited; the columns are:

1. citing: the patent on which the citation is found
2. cited: the patent that is cited.
3. cite_origin: whether the citation was supplied by the applicant (labeled ‘a’) or examiner (labeled ‘e’).
4. data_source: whether the citation information originated in the USPTO database (‘u’), in the EPO’s database (‘e’), or in the PCT database (‘w’).

### Patent classifications

**Main classification file.** File Name: Patent_classes.txt (Data Citation 1)

This is a harmonized list of patent classifications on all levels of resolution using the data we had available. USPTO patents in the Dataverse^15^ databases had US Patent Classifications (USPCs), while EPO and PCT had International Patent Classifications (IPCs) and Cooperative Patent Classifications (CPCs). The concordance between the two classification systems is provided in the USPC-IPC correspondence file (described below). 3 columns, 79.6 M lines, ‘|’ delimited; the columns are:

1. Pub_number: the patent publication number.
2. class: the patent classification number.
3. class_type: the type of classification this represents. ‘u’ indicates USPC, ‘i’ indicates IPC, ‘c’ indicates CPC, ‘m’ indicates an inferred IPC from the USPC-to-IPC concordance (described below), and ‘w’ indicates the WIPO field (described in the Main classification file (described above)).

**USPC-IPC correspondence.** File Name: USPC_to_IPC_fullList.txt (Data Citation 1)

The USPC and IPC systems were harmonized using the concordance provided by ReedTech at

http://patents.reedtech.com/classdata.php. In order to simplify the classification of patents, we compared all IPCs found in the OECD’s database to the USPTO classes liked in ReedTech’s concordance table and assigned them to the appropriate USPTO label. 2 columns, 183 K lines, ‘|’ delimited; the columns are:

1. USPC: the USPC
2. IPC: the IPCs that are found in our database that are equivalent to that USPC. If more than one IPC is equivalent, they are separated by a ‘,’.

**IPC-field correspondence.** File Name: IPC_to_Industry_WIPOJan2013.txt (Data Citation 1)

A linking between IPCs and coarse Sector and Field data, as defined in the Schmoch report^37^. 3 columns, 754 lines, tab delimited; the columns are

1. IPC_code: the IPC code
2. Sector_en: the top level sector classification.
3. Field_en: the top level field classification.

### Triadic patent families

File Name: patent_to_triadic_family.txt (Data Citation 1)

A linking between all EPO, USPTO, and PCT patents and the triadic families indicated by the OECD data. Two columns, 3.2 M lines, ‘|’ delimited; the columns are

1. Pub_number: the patent’s publication number
2. Triadic_Family: the triadic family in which the patent is included.

### New benchmarks

**Harvard inventors.** File Name: combined_labels_HarvardInv.txt (Data Citation 1)

The manual Harvard disambiguation used as one of the benchmarks in the paper. In all columns, if more than one ID is found, the elements are comma separated. 6 columns, 587 rows, ‘|’ delimited; the columns are:

1. Pub_number: the publication number of the patent
2. ManualIDs: The manually disambiguated IDs of each inventor on the patent.
3. OurIDs: the output of our algorithm on this patent.
4. LiLow: The ‘low’ disambiguation of Li *et al*. on this patent.
5. LiHigh: the ‘high’ disambiguation of Li *et al*. on this patent.
6. RawNames: the undisambiguated names on the patent, with case and punctuation dropped.

### Boston-area assignee benchmark

File Name: combined_labels_boston.txt (Data Citation 1)

The manual disambiguation of EPO/PCT assignees in the Boston area, used as one of the benchmarks in the paper. In all columns, if more than one ID is found, the elements are comma separated. 5 columns, 22528 rows, ‘|’ delimited; the columns are

1. Pub_number: the publication number of the patent
2. ManualIDs: The manually disambiguated IDs of each inventor on the patent. Each ID has the following structure: (1) a leading character of ‘r’, indicating within-region, or ‘e’, indicating an external collaborator; (2) a number with no specific meaning; (3) an ‘_’; and (4) a manual classification of the type of institution. The institution can have the values ‘university’, ‘hospital’, ‘company’, ‘lab’, and ‘other’.
3. OurIDs: the output of our algorithm on this patent.
4. HanIDs: The IDs for this patent from the HAN databases.
5. RawNames: the undisambiguated names on the patent, with case, punctuation, and spacing dropped.

Note that there is no correspondence between the boston-area IDs and the paris-area IDs (described in the Paris-area assignee benchmark section (described below)). Do not attempt to mix the two disambiguations together as incorrect ID-clashes are possible.

### Paris-area assignee benchmark

File Name: combined_labels_paris.txt (Data Citation 1).

The manual disambiguation of EPO/PCT assignees in the Paris area, used as one of the benchmarks in the paper. In all columns, if more than one ID is found, the elements are comma separated. 5 columns, 18877 rows, ‘|’ delimited; the columns are:

1. Pub_number: the publication number of the patent
2. ManualIDs: The manually disambiguated IDs of each inventor on the patent. Each ID has the following structure: (1) a leading character of ‘r’, indicating within-region, or ‘e’, indicating an external collaborator; (2) a number with specific meaning; (3) an ‘_’; and (4) a manual classification of the type of institution. The institution can have the values ‘university’, ‘hospital’, ‘company’, ‘lab’, and ‘other’.
3. OurIDs: the output of our algorithm on this patent.
4. HanIDs: The IDs for this patent from the HAN databases.
5. RawNames: the undisambiguated names on the patent, with case, punctuation, and spacing dropped.

Note that there is no correpsondence between the boston-area IDs (described in 3.6.2) and the paris-area IDs. Do not attempt to mix the two disambiguations together as incorrect ID-clashes are possible.

## Technical Validation

### Inventor and assignee benchmarks

We will quantify the compatibility of a trial disambiguation to the ‘correct’ ground truth partition using four different indicators: splitting, lumping, precision, and recall. Splitting and lumping are two related statistics to measure the similarity between two partitions that have been previously used for inventors in the UPSTO^15^, which respectively estimate the number of patents that are missing in the trial disambiguation in comparison to the benchmark (i.e., a true inventor is split into multiple IDs in the trial) and the number of patents that are added in the trial disambiguation compared to the benchmark (i.e., multiple trial IDs point to the same true inventor). The splitting associated with trial ID *i* is determined by identifying the true ID that shares the most patents, *m*_*i*_; with $P_{i}^{t}$ the set of patents invented by *i* in the trial partition and $P_{m_{i}}^{T}$ the set of patents invented by *m*_*i*_ in the true disambiguation, the splitting is $s_{i}=\left|P_{i}^{t}\backslash P_{m_{i}}^{T}\right|$ (i.e., the number of patents in the trial that *are* associated with *i* but *not* with *m*_*i*_ in the true disambiguation). Lumping is similarly defined for the true ID *j* by identifying the trial ID *M*_*j*_ with which it shares the most patents, with the lumping of *j* given by $l_{j}=\left|P_{j}^{T}\backslash P_{M_{j}}^{t}\right|$ (the number of patents in the trial that *are* associated with *M*_*j*_ but *not* with *j*). To perform this matching, we identify the best link for the ID with the largest number of patents (*i* or *j*, depending on if we are computing splitting or lumping), then progressively search for the best matches for smaller IDs that have not been previously assigned. Rarely occurring IDs may not be assigned a best match using this procedure (if all potential matches were previously assigned), and in this case all occurrences of that ID will be treated as erroneous. The total splitting and lumping of the trial partition are given by

(1)split=∑isi∑i|Pit|lump=∑jlj∑j|PjT|,

which estimates the fraction of patent that suffer from a splitting or lumping error.

**Figure 5:**
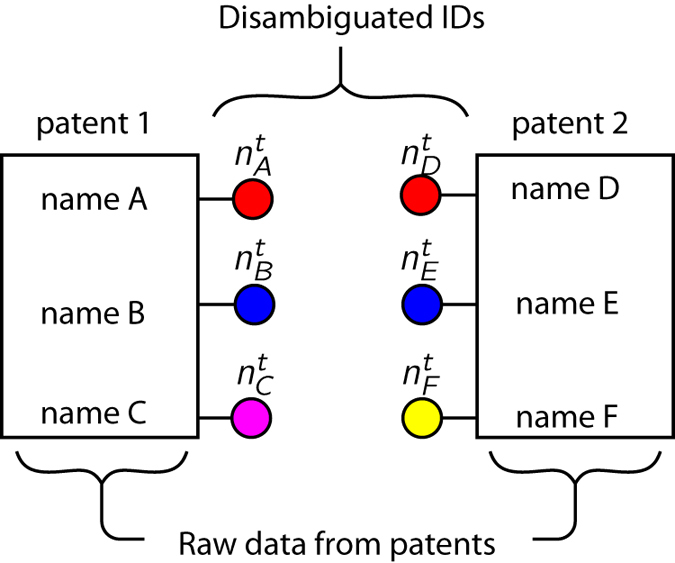
Schematic of the pairwise measurements of precision and recall. Each name is assigned a unique ID in the benchmark (indicated by the color of the circles) and a ID in the trial disambiguation (indicated by **n**^*t*^). Two true positives occur if $n_{A}^{t}=n_{D}^{t}$ and $n_{B}^{t}=n_{E}^{t}$. Two false negatives would occur if $n_{A}^{t}\neq n_{D}^{t}$ and $n_{B}^{t}\neq n_{E}^{t}$. A false positive occurs if $n_{C}^{t}=n_{F}^{t}$.

Splitting and lumping in equation (1) link trial IDs to true IDs that share the most patents in common, and treat patents assigned to a different ID as an error. While this is a useful statistic, it ignores the degree of splitting or lumping that has occurred: splitting remains unaltered by the number of IDs a true identity is associated with in the trial (and similarly for the lumping statistic), suggesting that some features of the similarity between partitions may be overlooked. We therefore also examine a pairwise measure of precision and recall of the trial and true disambiguations, schematically diagrammed in Fig. 5. The disambiguated name-to-ID’s $n^{T}(p)=\{n_{i}^{T}(p)\}$ for the *ith* name on patent *p* that can be compared to the disambiguated names in the trial disambiguation. For each pair of patents *p*_1_ and *p*_2_, we can determine the number of true positives, false positives, and false negatives between the trial and benchmark IDs by comparing the number of overlapping identifiers, as diagrammed in Fig. 5. Defining $x_{T}(p_{1},p_{2})=\left|\left\{n_{k}^{T}(p_{1})\cap n_{k}^{T}(p_{2}\right)\right|$ as the size of the intersection of the IDs in the benchmark and $x_{t}(p_{1},p_{2})=\left|\left\{n_{k}^{t}(p_{1})\cap n_{k}^{t}(p_{2}\right)\right|$ the size of the intersection in the trial disambiguation, there are at most *TP*(*p*_1_, *p*_2_)=min(*x*_*T*_, *x*_*t*_) IDs that agree in both partitions, *FN*(*p*_1_, *p*_2_)=max(0, *x*_*T*_−*x*_*t*_) matches in the benchmark not seen in the trial, and *FP*(*p*_1_, *p*_2_)=max(0, *x*_*t*_−*x*_*T*_) matches in the trial partition that don’t match in the benchmark. An estimate of the precision and recall for the trial is then

(2)prec=∑p1≠p2TP(p1,p2)∑p1≠p2[TP(p1,p2)+FP(p1,p2)]rec=∑p1≠p2TP(p1,p2)∑p1≠p2[TP(p1,p2)+FN(p1,p2)]

Note that this method does not compare the positions of the name matches (only the number of matches), and thus neglects the possibility of a transposition of the IDs in the trial (e.g., if names A and E are incorrectly linked together and *simultaneously* names B and D are incorrectly linked together). Due to the large number of IDs for both assignees and inventors, this type of error is expected to have a negligible effect. This approximation can be relaxed, at an increased computational cost. Using the definitions in equation (2), if all matches found in the trial are also found in the benchmark, the trial disambiguation will have high precision; and if all matches in the benchmark are found in the trial, the trial disambiguation will have high recall. These definitions of precision and recall are independent of a matching between the two partitions (that is, associating each trial ID with a ‘best’ true ID), comparing all assignments on an equal footing (but at greater computational cost due to the pairwise comparisons required).

In order to evaluate the accuracy of our inventor disambiguation, it is necessary to find a set of ground truth identification of patent inventors: a manually curated subset of the patent data for which a correct disambiguation of the names has been performed. Such a benchmark exists for ~100 USPTO inventors of ~1300 patents in the area of engineering and biochemistry that has been used as a golden standard in the literature^15^. This is a complete disambiguation of all patents invented by a specific set of inventors. We also generated a manual disambiguation of USPTO patents filed by assignees containing the phrases ‘harvard college’ and ‘harvard university’ (case insensitive) in the name. This second benchmark is a complete disambiguation of all inventors on a specific set of patents, with 587 patents with 1,000 name/address pairs and 827 disambiguated inventor IDs (using our approach). In Tables 4 and 5, we compare the splitting, lumping, precision, and recall of four potential disambiguations: ‘none’ (meaning that case and punctuation are ignored but no further disambiguation is performed), ‘lower’ and ‘upper’ (two outputs of the disambiguation of Ref 15), and our approach. For the golden standard benchmark (Table 4), all patents linked to a trial ID are included when computing the statistics (including incorrectly-assigned patents, representing false positives), but un-disambiguated collaborators of these inventors are ignored. For the Harvard inventor disambiguation shown in Table 5, only the disambiguated patents are included (and no additional patents that may represent false positives are added) because we do not have a complete list of the ‘correct’ patents for each inventor. The exclusion of such false positives increase the apparent precision of each method in Table 5, since exact name matches for inventors affiliated with Harvard are very likely referring to the same person.

In the left side of Tables 4 and 5, we see that all disambiguation methods (‘lower,’, ‘upper,’ and ‘ours’) provide an improvement over the un-disambiguated results in all statistics, and in general the results of Li *et al*.^15^ produce a higher improvement in all statistics in comparison to our approach. The quality of our disambiguation is comparable to that of Li’s in the case of the Harvard dataset, but in the case of the Golden Standard benchmark our results have about half the lumping of the un-disambiguated name and major improvements in both precision and recall. The disambiguation of Li is a more significant improvement over the raw names in lumping, splitting, and recall, but is comparable to our approach in precision. A higher recall can be found by removing all IDs associated with low-quality disambiguations in our approach (those IDs that did not incorporate correction of spelling errors in steps 1 and 5 of Fig. 4). On the right sides of Tables 4 and 5, we discard all patents having at least one low-quality ID, as well as all names or Li IDs occurring on those patents. We find a significant improvement in all quality statistics using our approach in both benchmarks, although still marginally underperforming Li *et al.* in both datasets. These results indicate that our inventor disambiguation is expected to have high precision in all cases (i.e., will have very few false positives), but higher recall (very few false negatives) for high-quality disambiguated names. Low-quality disambiguations are expected to underestimate the number of patents invented by the person, but not significantly overestimate them.

We are not aware of a freely available ground truth disambiguation of assignees, and in order to test the accuracy of our methods we manually generated our own benchmark from a by-hand disambiguation of a subset of patent assignees in the EPO and PCT data. To create a benchmark of manageable size, we focused on assignees in the EPO and PCT in specific regions active in specific fields of research. The OECD REGPAT database^27^ provides geolocation information on the level of NUTS3, and we generated a list of all assignees on patents assigned to names in Boston or Paris (the selection of assignees was made where Boston assignees have addresses in the NUTS3’s US25017, US25025, or US25021 and Paris have addresses in NUTS3’s beginning with ‘FR10’ in the OECD RegPat database^27^. We note that the OECD’s geolocation process is independent from our own, so this benchmark is not affected by any potential errors or incompleteness in our geolocation procedure.). These names include both regional assignees as well as external collaborators of those regional institutions, and in order to reduce the number of assignee names to disambiguate, we retained only assignees names found on at least ten biopharma patents (biopharma patents are defined as having an IPC classification which falls under the fields ‘Pharmaceuticals’ or ‘Biotechnology’ using the WIPO field-level aggregation of patent classes^37^). Exclusion of less-common names greatly reduced the number of manual matches that were required, but may introduce a bias against small startups, individuals that were assignees, or large institutions that rarely work in the biopharma fields. This produced a final set of ~700 disambiguated assignees (from ~900 raw names) on ~23 K biopharma patents in the Boston area and 640 disambiguated assignees (from ~1,100 raw names) on ~19 K biopharma patents in the Paris area.

In Table 6, we determine the error statistics for undisambiguated names (removing spacing and punctuation); the HAN name harmonization, which accounts for synonyms such as ‘Co’ and ‘Company’ in assignee names (with unique HAN IDs extracted directly from the OECD’s HAN database^23^); and the method in this paper (generated using the OECD’s REGPAT database^27^). We are not aware of another freely available assignee disambiguation of EPO and PCT patents that can be used for comparison. As was the case in Table 5, false positives from patents not found in the manual disambiguation are excluded from this trial, and the precision is likely to be inflated (because our approach permits a maximum distance between geolocations of 20 km on the disambiguation of assignees, it is unlikely that the precision using our approach is significantly higher than what would be observed if all patents were included.). In all cases the HAN harmonization is a marginal improvement over the raw names, but our method produces a significant improvement over both in every statistic. The lower overall recall in the Paris area is due to many alternate spellings from the presence or absence of accents on words, a complication not typically present in the Boston area. Note that the names in the Paris area involve terms in French (e.g., ‘societé aononyme’ is a commonly occurring substring of assignee names), and the high precision of our disambiguation in the Paris area suggests that the algorithm is robust to variations in language. Over 95% of the patents in Table 6 are assigned to solely high-quality assignee disambiguations, and there is little benefit in dividing the results into differing qualities of disambiguation (as was beneficial in Tables 4 and 5). The error statistics in Table 6 suggest that our assignee disambiguation is likely reliable in many major global research hubs.

### Leading inventors and assignees

Bibliometric data has been implemented in a variety of contexts in both publications^7,11,38^ and patents^2,4,10,24,25,39^ to track individual careers, institutional collaborations, and information flows between regions. In almost all studies, major players represented by top authors, inventors, or institutions tend to be emphasized (as measured by the number of patents or publications, number of citations, or other quality indicators), due to their high productivity and output. It is therefore worthwhile to determine how accurately our approach is able to identify the top players in the world, and what advantages our method may have over existing datasets.

In Table 7, we list the top ten players as determined by Li’s ‘lower’ inventor disambiguation of USPTO inventors (the ‘upper’ disambiguation does not differ significantly on these names) compared to the output of our methods. We find a negligible difference (typically less than 2% variation in the patent counts) between the approaches in the majority of cases, but significant differences do sometimes occur. In the case of Gurtej Sandhu, we find our algorithm detects an additional ~100 patents, an improvement over Li’s approach sufficient to change his ranking from 7*th* to 5*th* place in our ordering. Our method sometimes does underestimate the patents invented by top players, particularly for Japanese inventors. This is due to the low-quality of geolocations provided by YQL in Japan: of the ~100 K address containing the word ‘Tokyo,’ 0.6% produced a high-resolution geolocation (meaning that virtually no Japanese inventor will have a high-resolution geolocation) but 82% are found on the neighborhood level or better (meaning they contained more information than simply a city name). It may therefore be reasonable to expect a higher number of splitting errors for Japanese inventors using our data. Table 7 indicates that, with the exception of these splitting errors of Japanese inventors, our method is able to reliably detect prolific inventors consistent with Ref. 15.

A surprising aspect of Table 7 is that not a single European is found in the list of the top 20 most prolific inventors. This is due to an intrinsic bias in the choice of USPTO patents: Europeans may be more likely to apply for patents in the EPO (which grants protection in Europe where they live and work) than in the USPTO. Non-US and non-European inventors and assignees may likewise be more likely to submit patents to under the PCT than in either the USPTO or EPO, to ensure international and domestic protection of their inventions. A significant advantage of our method is that these three major offices are linked in the disambiguation, permitting a more even accounting of highly prolific inventors. In Table 8, we see that while the top five inventors worldwide all focus their patenting activity most heavily in the USPTO, many prolific inventors focus their patent applications in the EPO or under the PCT. We are able to identify a number of top inventors from China (filing their patents almost exclusively under the PCT) and Germany (filing their patents in both the EPO and USPTO, typically), many of whom would not be seen in the top hundred inventors when focused solely on the USPTO. When these disambiguated IDs are low resolution, we continue to expect most errors to be splitting (thus underestimating the total number of patents invented by that person) instead of lumping (which would overestimate that person’s patent count). Table 8 suggests that our inventor disambiguation may be more appropriate in studying the effects of global knowledge production and international knowledge spillovers through collaborations across the Atlantic as well as in emerging economies.

It is also useful to compare our disambiguation to that of Li *et al.*^15^ on names that are in some sense difficult, rather than solely on the most prolific 20 inventors. To do so, we also extract the top 20 disambiguated inventors in the USPTO using our algorithm with a two- or three-character last name. These last names tend to be of Asian descent and to be more common globally than long names are, and we might naturally expect a greater level of lumping errors in these names than the (generally more European) names in the top 20 globally prolific inventors. In Table 9, we compare our IDs to those of of Li *et al.*, and examine the level of overlap between the two disambiguations. In 75% of the cases, there is a negligible difference between the two disambiguations, and a ≥95% overlap between our disambiguation and the most common shared ID in Li’s disambiguation.

The column ‘diff’ in Table 9 indicates the apparent meaning of the difference between our disambiguation in the 4 inventors for which our disambiguation differs significantly from Li’s. It takes on one of three values, denoting the apparent correctness of differences between our disambiguation and Li’s: ‘−’ denoting a negligible difference (16 of the 20 cases), ‘G’ denoting an apparent gain of information by connecting distinct IDs in Li’s disambiguation that likely refer to the same person (3 of 20 cases), and ‘L’ an apparent loss of information by apparently erroneously connecting IDs in Li’s disambiguation that are probably correctly separated (1 of 20 cases). In two cases, the gains are due to the flexibility of our name matching algorithm, which overcomes difficulties in linking identities with multiple and variable middle names (Louis Lu Chen Hsu and Chen Hua Douglas Yu). The two other differences between our disambiguation and that of Li’s are due to our linking of exact name matches with other characteristics in common (citations, coinventorships, or assignees), leading to the beneficial merging in the case of Jerry Wu (working with assignee ‘Hon Hai Precision Ind. Co. Ltd.’ in both locations), and to the apparently detrimental clustering in the case of Hiroshi Ito (simultaneously working in multiple cities and with multiple different assignees). Lumping errors are thus possible using our algorithm (as they are in any algorithm), but we see no evidence of a systematic inflation of the patenting of short, commonly occurring names. Rather, Table 9 indicates that our approach is consistent with existing disambiguations and in some cases may improve the name disambiguation due to improvements with the flexible name matching.

Another significant advantage of our methodology is the simultaneous disambiguation of assignees, allowing us to determine the major research institutions in addition to prolific inventors. In Table 10, we see the top patent assignees are dominated by computer and communications companies, with all members of the list firms with highly recognizable names. We also list the number of unique names that were involved in the disambiguation (spacing and punctuation ignored), highlighting the extreme variability of names that may occur. US and Japanese firms tend to patent in the USPTO (as was the case for inventors), and most European countries tend to patent more heavily in the EPO. The exceptions of Nokia Siemens in Finland and Ericsson in Sweden, who tend to evenly split their applications between the EPO and under the PCT, may be explained by these countries joining the European Union in 1995 (note that while Sweden joined the EU in 1995, it was a signatory of the European Patent Convention in 1978.). Note also that Nokia Siemens is found twice in this list: once in Germany and once in Finland, with the appropriate designations of a limited liability corporation (‘GMBH’ and ‘OY’, respectively) in their countries. Our method correctly treats these as distinct entities despite their similar names, due to the 20 km geolocation restriction on assignees. Note that in order to determine the number of patents held by a multinational corporation (in the case of Nokia Siemens, the NOKIA group), an ownership tree must be determined from some alternate datasource. All of the European firms in Table 10 would see their ranking drop significantly if only the USPTO were taken into account, with Nokia-Siemens’ German office the only EU research lab that would be included in the top 20. As was the case for inventors, by including the USPTO, EPO, and PCT, a broader, more international picture of major research institutions can be extracted than if only one were included.

Our assignee disambiguation algorithm searches for similar names at identical positions and identical names at nearby positions, linking regional elements together that represent the same institution (as illustrated in Fig. 2). The accuracy of our approach and the difficulty in such a disambiguation is shown in Table 11, where the commonest names associated with the NIH are shown (and correctly linked to one another). While many have a similar structure between them (i.e., text indicating US government, followed by text indicating department), there is significant heterogeneity in the names that would make simple string matching difficult. Linking the name ‘National Institutes of Health’ to the other names in this list would likely be unfeasible unless a high-resolution geolocation approach were used, as the only word shared between the names is ‘health,’ commonly occurring in the region. We note that there are also a large number of other government offices within 20 km having a similar naming structure to the NIH (and to each other), and our algorithm correctly distinguishes between them.

Government labs and universities tend to have a relatively simple structure, operating in a single region. Multinational corporations have a more complex and non-regional structure, and it is worth emphasizing what our disambiguation is actually identifying in the case of companies. A disambiguation of assignees for heterogeneous entities like IBM can potentially be performed on two levels: a disambiguation of the individual elements on a regional level, or a disambiguation of the full legal structure of the entity regardless of proximity. Our algorithm performs the former: we have have disambiguated extremely heterogeneous names that refer to the same regional entity, with global coverage. Linking these regional entities under their correct global corporate umbrella is a valuable exercise^23^, but not attempted here. Further work can be employed using this local disambiguation coupled with other databases, including ownership databases or wikipedia, to link regional entities to their corporate owners. Examples of the meaning of the disambiguated assignees for two major corporations (IBM and Nokia Siemens) are presented in Table 12, and highlight the fact that the individual disambiguated entities are meaningful, referring to different research labs or production facilities.

The assignee name ‘International Business Machines Corporation’ is the second most commonly occurring in the patent database, and while it is almost certainly the case that each of these patents were ultimately developed under the direction of IBM, the location where the work was actually done may vary. IBM’s main offices and labs are in Armonk NY, but semiconductor design was worked on in Fishkill NY and its first manufacturing plant was in Endicott NY. Table 12 highlights the fact that, rather than focused on a single legal entity regardless of geography, our algorithm identifies productive sub-units of those legal entities. Rather than aggregating each ‘international business machines’ lab into a single corporate entity, our algorithm is identifying the regional players, agnostic to the corporate structure that may be directing the research activities of those labs. We note the algorithm is note immune from errors: HA5998 is not aggregated into HA1, the main IBM ID, due to a spelling error in the city. The address fields for HA5998 read ‘Armond NY’ rather than ‘Armonk NY’, which yahoo’s geolocation placed more than 20 km distant. This erroneous disambiguation represents a splitting error well below 1%, showing the algorithm is still quite robust.

Nokia Siemens has the second most prolific regional lab in the world (using our algorithm), with the main lab in Munich having over 95% of its patents under the original name of Siemens AG. Importantly, our algorithm is able to correctly link patents produced at this location after the merger of Nokia and Siemens: every occurrence of the name ‘Nokia Siemens Networks GMBH’ is also (correctly) linked to the Nokia Siemens disambiguated ID HA2. Likewise, the names associated with HA15, the main Nokia Siemens lab in Helsinki, are composed of ~90% ‘Nokia Corp’ and ~10% ‘Nokia Siemens Networks OY.’ This implies that patents are linked to the disambiguated regional institution both before and after the two companies merged. Abrupt name changes in a company (as may happen due to a merger) will prevent our algorithm from preserving the links between that institution’s patents after the change. Drastic name change events can only be fully accounted for using external data sources that track these legal events.

## Additional Information

**How to cite this article:** Morrison, G. *et al.* Disambiguation of patent inventors and assignees using high-resolution geolocation data. *Sci. Data* 4:170064 doi: 10.1038/sdata.2017.64 (2017).

**Publisher’s note:** Springer Nature remains neutral with regard to jurisdictional claims in published maps and institutional affiliations.

## Supplementary Material



Supplementary Information

## Figures and Tables

**Table 1:** A comparison between different existing disambiguation algorithms covering patent data.

**Characteristic**	**Li**^15^	**Raffo**^21^	**Pezzoni**^17^	**Ventura**^22^	**HAN**^23^	**Ours**
Covers USPTO inventors	✓	Partial		Partial		✓
Covers EPO/PCT inventors		Partial	✓			✓
Covers assignees			✓		✓	✓
Rule/threshold algorithm	✓	✓	✓		✓	✓
Supervised learning				✓		
Uses string matching	✓	✓	✓	✓	✓	✓
Uses co-inventorship	✓	✓	✓	✓		✓
Uses co-assignment	✓	✓	✓			✓
Uses assignee legal status					✓	
Uses patent citations	✓		✓			✓
Uses patent classes	✓		✓	✓		
Uses location data	✓	✓	✓	✓		✓
Uses street-level geolocation						✓
Disambiguation data output freely available	✓				✓	✓
The first block of characteristics indicates the coverage or type of algorithm, the second block indicates the information used by the algorithm, and the third block indicates the status of a free and complete data release. ‘Partial’ indicates the algorithms were applied to a relatively small but curated subset of the patent data.						

**Table 2:** Summary of the assignee and inventor coverage of the disambiguation in the three patent offices (patent count in millions).

	**EPO**	**PCT**	**USPTO**
Assignee Disambiguation statistics			
Patents with Assignees	2.68	2.36	3.61
Patents with assignee addresses	2.68 (100%)	2.36 (100%)	2.01 (56%)
Patents linked to geolocated IDs	2.62 (99%)	2.28 (97%)	3.18 (88%)
Patents with all high-qual disambiguated IDs	1.95 (73%)	1.44 (61%)	1.89 (52%)
Inventor disambiguation statistics			
Patents with inventors	2.68	2.34	4.24
Patents with inventor addresses	2.67	2.27	4.24
Patents linked to geolocated IDs	2.62 (98%)	2.24 (96%)	4.15 (98%)
Patents with all high-qual disambiguated IDs	2.05 (78%)	1.34 (57%)	2.09 (49%)
Listed are the number of patents having an assignee or inventor listed in the raw data (note that these fields may be blank); the number of patents with any address; the number of patents for which all of those entities is linked to a geolocated and disambiguated ID (of either high or low quality); and the number of patents for which all of those entities are high-quality (the disambiguation involved both geospatial information as well as noise correction in the name).			

**Table 3:** Summary of the total coverage of the disambiguation in the three patent offices (patent count in millions).

	**EPO**	**PCT**	**USPTO**	**Tot**
Patents in database	2.68	2.36	4.24	9.29
Patents with all names located & disambiguated	2.58 (98%)	2.22 (98%)	3.76 (89%)	8.53 (92%)
Patents with all high-quality disambiguations	1.68 (63%)	1.00 (42%)	1.26 (30%)	3.94 (42%)
Listed are the number of patents having an assignee or inventor listed in the raw data (note that these fields may be blank); the number of patents with any address; the number of patents for which all of those entities is linked to a geolocated and disambiguated ID (of either high or low quality); and the number of patents for which all of those entities are high-quality (the disambiguation involved both geospatial information as well as noise correction in the name).				

**Table 4:** Benchmarking of inventor disambiguations on the Golden Standard benchmark.

**Gold Standard Inventor Disambiguation**				
	**None**	**Lower**	**Upper**	**Ours**
All patents				
# pats	1582	1320	1320	1321
# IDs	176	118	121	150
lumping (lower better)	29.8%	4.9%	5.3%	10.5%
splitting (lower better)	15.2%	3.8%	4.2%	9.5%
precision (higher better)	0.50	0.97	0.97	0.94
recall (higher better)	0.36	0.88	0.88	0.82
Only high-quality IDs				
# pats	825	606	606	608
# IDs	88	58	59	62
lumping (lower better)	30.4%	2.0%	2.6%	4.1%
splitting (lower better)	10.5%	1.5%	2.0%	4.1%
precision (higher better)	0.22	0.97	0.97	0.98
recall (higher better)	0.23	0.97	0.96	0.92
The column ‘none’ refers to a name disambiguation that simply removes punctuation and differences between upper and lowercase letters, the columns ‘lower’ and ‘upper’ refer to the two disambiguations of Li *et al.*^15^, and the column ‘ours’ refers to this work. In the table on the left, the benchmark includes all patents found in the Golden Standard as well as all USPTO patents in the disambiguations with at least one trial ID (thus including patents erroneously assigned to the disambiguated IDs). In the table on the right, the dataset is restricted only to patents for which our disambiguation has all ‘high-quality’ IDs, for which both geolocation and spelling errors were potentially corrected.				

**Table 5:** Benchmarking of inventor disambiguations on patents assigned to Harvard.

**Harvard Inventor Disambiguation**				
	**None**	**Lower**	**Upper**	**Ours**
All patents				
# pats	587	587	587	587
# IDs	877	827	829	827
lumping (lower better)	4.0%	0.7%	0.7%	1.6%
splitting (lower better)	3.6%	0.7%	0.9%	1.5%
precision (higher better)	1.00	1.00	1.00	1.00
recall (higher better)	0.89	0.96	0.96	0.96
Only high-quality IDs				
# pats	115	115	115	115
# IDs	177	165	165	164
lumping (lower better)	5.6%	0.2%	0.3%	0.0%
splitting (lower better)	4.5%	0.3%	0.3%	0.0%
precision (higher better)	1.00	1.00	1.00	1.00
recall (higher better)	0.86	0.99	0.99	1.00
The columns are the same as in Table 4.				

**Table 6:** Benchmarking of assignee disambiguations in the EPO and under the PCT for assignees geolocated in the Boston (left) or Paris (right) areas.

	**None**	**HAN**	**Ours**
Boston Area Assignees			
# pats	22527	22175	22527
# IDs	376	691	357
lumping (lower better)	19.0%	11.5%	4.0%
splitting (lower better)	15.5%	12.8%	3.5%
precision (higher better)	1.00	0.99	1.00
recall (higher better)	0.74	0.76	0.99
Paris Area Assignees			
# pats	18876	18011	18876
# IDs	279	480	248
lumping (lower better)	34.0%	23.8%	8.0%
splitting (lower better)	26.4%	25.3%	8.0%
precision (higher better)	1.00	0.97	0.97
recall (higher better)	0.51	0.53	0.95
The column ‘none’ refers to a name disambiguation that simply removes punctuation and differences between upper and lowercase letters, the column ‘HAN’ refers to OECD’s Harmonized Assignee Name identifier, and the column ‘ours’ refers to this work.			

**Table 7:** The top ten patent inventors by number of patents in the USPTO (1980–2010), ranked using the disambiguation of Li *et al.*^15^ and ours (matching IDs between the two algorithms were based on the number of shared patents).

**USPTO rank (Li)**	**Name**	**Country**	**USPTO (Li**^15^)	**USPTO (ours)**	**rank (ours)**	**% diff (high qual)**	**% diff (low qual)**
1	Kia Silverbrook	AU	3570	3560	1	−0.3%	
2*	Shunpei Yamazaki	JP	2519	2398	2		−4.8%
3	Donald Weder	US (IL)	997	998	3	+0.1%	
4	Leonard Forbes	US (OR)	932	932	4	0%	
5	Paul Lapstun	AU	866	871	5	+0.5%	
6	Chang Hwan Hwang	KR	840	838	7	−0.2%	
7	Gurtej Sandhu	US (ID)	782	869	6	+11%	
8	Warren Farnworth	US (ID)	734	723	8	−1.5%	
9	Salman Akram	US (ID)	676	676	9	0%	
10*	Jun Koyama	JP	671	533	15		−21%
11	William Wood	US (CA)	647	649	10	+0.3%	
12	Austin Gurney	US (CA)	620	620	11	0%	
13	Audrey Goddard	US (CA)	606	606	12	0%	
14*	Akira Suzuki	JP	605	377	43		−38%
15	Paul Godowski	US (CA)	587	587	13	0%	
16	George Spector	US (NY)	556	553	14	−0.5%	
17	Mark Gardner	US (TX)	521	516	16	−1.0%	
18	Simon Walmsley	AU	511	512	17	+0.2%	
19	Jay Walker	US (CT)	509	510	18	+0.2%	
20*	Tetsujiro Kondo	JP	509	385	38		−24%
In the majority of cases our algorithm produces a nearly identical number of patents, but it performs worse on Japanese inventors that have many low quality geolocations. Inventor locations indicate the position of the majority of their patents, as most inventors provide addresses in multiple countries. The ‘% diff’ columns refer to the percent change in the number of patents assigned to that identity in our disambiguation relative to Li’s, and are divided between high quality (having both geolocation and spelling correction) and low quality (having only geolocation). IDs that are categorized as low quality using our approach are denoted by a *.							

**Table 8:** The twenty most prolific inventors in the combined USPTO, EPO, and PCT according to our disambiguation (recall that Table 7 included only USPTO patents), along with the specific counts per office.

**Total rank**	**Total pats**	**Name**	**Country**	**USPTO rank**	**USPTO pats**	**EPO rank**	**EPO pats**	**PCT rank**	**PCT pats**
1	4623	Kia Silverbrook	AU	1	**3560**	2	523	3	540
2*	2503	Shunpei Yamazaki	JP	2	**2398**	—	83	—	22
3	1261	Paul Lapstun	AU	5	**871**	102	189	62	201
4	1260	Tadahiro Ohmi	JP	20	**472**	11	356	6	432
5	1244	Eberhard Ammermann	DE	25	447	1	**541**	33	256
6	1085	Donald Weder	US (IL)	3	**998**	—	44	—	43
7*	1075	Yi Xie	CN	—	11	—	3	2	**1061**
8*	1075	Yumin Mao	CN	—	12	—	2	1	**1061**
9	1067	Craig Rosen	US (MD)	81	298	9	378	10	**391**
10	1052	Steven Ruben	US (MD)	—	245	6	390	7	**417**
11	1024	Gurtej Sandhu	US (ID)	6	**869**	—	57	—	98
12	1019	Leonard Forbes	US (OR)	4	**932**	—	37	—	50
13	1014	William Wood	US (CA)	10	**649**	48	244	—	121
14	977	Austin Gurney	US (CA)	11	**620**	53	236	—	121
15	955	Heinz Focke	DE	24	**448**	3	441	—	66
16	906	Audrey Goddard	US (CA)	12	**606**	82	206	—	94
17	897	Siegfried Strathmann	DE	—	176	10	**372**	14	349
18*	870	Akira Suzuki	JP	43	**377**	13	331	—	162
19	850	Chang Hwan Hwang	KR	7	**838**	—	12	—	0
20	846	Paul Dent	US (NC)	60	**331**	30	269	38	246
Inventors rankings in each office are suppressed if they are not in the top 100 most prolific inventors in that office. The most common office to which each inventor applies is in bold face text. IDs that are categorized as low quality using our approach are denoted by a*.								

**Table 9:** Comparison of our disambiguation to that of Li *et al.*^15^, restricted the 20 most prolific USPTO inventors with at most three letters in their last name.

***n***	**US patents**	**Diff**	**Our ID**	**Li ID%**	**Name**	**Location**
1	493	—	HI105	03895147-1100	AHN, KIE Y	CHAPPAQUA NY US
2	463	**G**	HM28	04758531-261	HSU, LOUIS L	FISHKILL NY US
				05384152-225	HSU, LOUIS LU CHEN	FISHKILL NY US
				06864540-113	HSU, LOUIS C	FISHKILL NY US
3	409	—	HM181	05304728-1100	EBY, WILLIAM H	PANORA IA US
4	381	—	HM85	05555157-2100	IVE, JONATHAN P	SAN FRANCISCO CA US
5	373	—	LM52	06150701-197	LEE, CHANG SOO	INCHEON KR
6	356	—	HX152	03946327-1100	HSU, SHENG TENG	CAMAS WA US
7	322	—	LI106	05083197-1100	KO, JUNG WAN	YONGIN KR
8	308	**G**	LM228	06565366-186	WU, JERRY	IRVINE CA US
				05820403-412	WU, JERRY	CHANG-HUA HSIEN TW
9	300	—	HM224	05880511-1100	YU, BIN	CUPERTINO CA US
10	296	—	LI149	05825103-1100	LEE, KYUNG GEUN	KYONGGI-DO KR
11	291	**L**	LI119	03920102-132	ITO, HIROSHI	TOKYO JP
				04129051-231	ITO, HIROSHI	TOYOTA JP
				04436313-48	ITO, HIROSHI	TSURUGASHIMA JP
				06697579-16	ITO, HIROSHI	OSAKA JP
				06697579-15	ITO, HIROSHI	MOBARA JP
12	271	—	HX366	05736423-1100	NGO, MINH VAN	FREMONT CA US
13	264	—	HI675	04109082-1100	SIH, JOHN C	KALAMAZOO MI US
14	224	—	LM496	06049450-2100	KIM, NAM MI	SEOUL KR
15	218	**G**	LM739	05518959-291	YU, CHEN HUA	HSIN-CHU TW
				05654233-18	YU, CHEN HUA DOUGLAS	HSIN-CHU TW
16	215	—	HI877	04125338-179	LEW, HYOK S	ARVADA CO US
17	214	—	HX214	04514144-198	LEE, CHING PANG	CINCINNATI OH US
18	213	—	HM368	06354505-3100	ZHU, XIAOXUN	MARLTON NJ US
19	211	—	HI116	04621337-2100	COK, RONALD S	ROCHESTER NY US
20	210	—	LM636	05407364-399	WU, KUN TSAN	TUCHENG TW
*n* denotes the rank of the inventor in this list, followed by the number of USPTO patents attached to our inventor ID. ‘diff’ denotes the difference between our disambiguation for this inventor and the disambiguation of Li, described in the text. Our IDs may be linked to one or more of Li’s IDs, and the fraction of patents linked to our ID also linked to that Li ID is in the 6th column. We do not list IDs with overlap below 5%.					

**Table 10:** The twenty largest assignee headquarters as measured by the total number of patents worldwide, 1980–2010, along with the nation of the geolocation and the number of names for that assignee that were used in the disambiguation.

**Total rank**	**Total pats**	**Name**	**Country**	**# clean names**	**# Rawnames**	**USPTO rank**	**USPTO pats**	**EPO rank**	**EPO pats**	**PCT rank**	**PCT pats**
1	73586	IBM	US (NY)	20	49	1	**53027**	7	15320	23	5239
2	66518	Nokia Siemens	DE	30	52	15	14439	1	**33857**	2	18222
3	65356	Phillips	NL	13	44	25	9959	2	**32084**	1	23313
4	56481	Cannon	JP	1	7	2	**38483**	6	15359	54	2639
5	49288	Robert Bosch	DE	12	24	21	11979	3	**20624**	3	16685
6	45634	Matsushita Electric	JP	14	84	4	**24867**	8	14401	17	6366
7	44663	Sony	JP	5	13	5	**23783**	5	15626	22	5254
8	39147	NEC	JP	3	6	7	**22739**	14	10793	21	5615
9	38759	Samsung	KR	18	93	3	**25922**	12	10912	80	1925
10	37306	BASF	DE	10	20	28	9448	4	**17475**	7	10383
11	34842	Hitachi	JP	5	32	6	**23661**	24	7832	37	3349
12	34225	Gen. Electric	US (NY)	13	30	10	**18633**	9	12306	38	3286
13	31340	Eastman Kodak	US (NY)	5	11	12	**16672**	16	10628	28	4040
14	30076	Nokia Siemens	FI	26	62	39	7554	13	10871	6	**11651**
15	28194	Proctor & Gamble	US (OH)	18	51	34	8401	17	**10510**	9	9283
16	27870	Ericsson	SE	16	68	64	5118	15	10662	5	**12090**
17	27837	Intel	US (CA)	9	21	9	**18688**	62	3506	20	5643
18	27442	Du Pont	US (DE)	20	92	23	**10757**	19	9601	13	7084
19	26309	Toshiba	JP	2	8	18	**12342**	10	11322	53	2645
20	26128	Microsoft	US (WA)	6	16	13	**16629**	35	5384	26	4115
The column ‘# clean names’ refers to the number of unique names with geolocations, ignoring spacing, capitalization and punctuation, while the ‘raw’ names are the count of unaltered strings referring to that institution. Also shown are the patent counts in the various offices as well as their rank in those offices. Many major assignees focus their patenting applications on the EPO or under the PCT, and a focus solely on USPTO would overlook major players in the global innovation network.											

**Table 11:** Names appearing on at least 100 patents that are aggregated into a single entity for the NIH in Bethesda MD using our algorithm.

**ID**	**#**	**Name**
HA160	865	the united states of america as represented by the department of health and human services
	681	the united states of america as represented by the department of health
	627	the government of the united states of america as represented by the secretary of the department of health and human services
	420	the government of the united states of america, as represented by the secretary, department of health and human services
	259	the government of the united states of america, as represented by the secretary of the department of health and human services
	234	the government of the united states of america, as represented by the secretary, department of health and human services
	204	the united states of america as represented by thesecretary of the department of health and human services
	173	the u.s.a. as represented by the secretary, department of health and human services
	154	the united states of america, as represented by the secretary, department of health and human services
	143	the government of the united states of america as represented by the secretary of the department of health and human services
	106	national institutes of health
HA108	7680	the united states of america as represented by the secretary of the navy
HA314	3418	the united states of america as represented by the secretary of the army
HA681	896	the united states of america as represented by the secretary of agriculture
In generating this table, the text was converted to lowercase, but spacing and punctuation were not altered. The top names are clearly related to one another and their location in Bethesda indicates the assignment to the NIH ID is reasonable. At the bottom of the table are listed the most common names for other US departments and the ID associated with each (none of which are matched to the NIH), despite the fact they are within the 20 km radius used in our algorithm with a large number of words in common.		

**Table 12:** The five most prolific assignees containing the string ‘international business machines’ (top) and either ‘nokia’ or ‘siemens’ (bottom) in our disambiguation.

ID	**Patents**	**Name**	**Location**
HA1	77111	International Business Machines Corporation	Armonk NY US
HA403	2439	International Business Machines Corporation	Fishkill NY US
HA814	1202	International Business Machines Corporation	Endicott NY US
HA2888	314	International Business Machines Corporation	Lexington KY US
HA5998*	136	International Business Machines Corporation	Armond NY US
HA2	74433	Siemens AG	Munich DE
HA15	33010	Nokia Corporation	Helsinki FI
HA84	8763	BSH Bosch und Siemens Hausgeräte GmbH	Munich DE
HA337	2840	Siemens AG	Nuremberg DE
HA552	1765	Siemens Automotive Corporation	Pontiac MI US
The assignee disambiguation does not resolve the various legal entities under a single umbrella (either nationally in the case of IBM or internationally in the case of Nokia Siemens), but rather focuses on individual subsidiaries that are located in different regions.			

*denotes a splitting error for the IBM dataset due to an alternate location spelling (described in the text).
