# Supplementary material for: Disambiguation of patent inventors and assignees using high-resolution geolocation data
Source: Sci Data. 2017 May 16;4:170064. doi: 10.1038/sdata.2017.64 (PMC5433392; doi:10.1038/sdata.2017.64)
Supplement: Supplementary Information [file sdata201764-s2.pdf]

Supplementary Information for 'Disambiguation of Patent Inventors and Assignees Using High-Resolution Geolocation Data'

G Morrison, M Riccaboni, and F Pammolli

April 3, 2017

1 Geolocation

Our disambiguation relies heavily on the geolocation of inventors and assignees using the addresses provided in the patent data. The names and addresses for each patent were extracted from the OECD Regpat January 2014 database [1] for the EPO and PCT patents, and from the patent database provided by Li et al [2] for the USPTO patents. The addresses are uploaded to yahoo's YQL API (with a UTF-8 encoding of the address; no additional cleaning performed), with a JSON response returned by the server containing a great deal of information about the geolocation(s) of that address. If the address was successfully located by YQL, we extracted all latitude and longitude data along with the quality of that geolocation, where the quality is an assessment of how precise the lat/long is (e.g. street level vs. city level). Note that in principle other geolocation APIs could be used (e.g. OpenStreetMap or Google places), but YQL was chosen due to its familiarity and ease of use. After the geolocation was complete on the January data, we acquired the July 2014 OECD Regpat database [3]. The geolcations extracted from the January database addresses were applied to the July database without modification, so some addresses from the newer data release may be missing.

The number of patents with geolocations are listed in Table 1, with high coverage of the geolocation for all inventors and all assignees *except* for the USPTO. 1,847,909 USPTO patents have absolutely no assignee address information (no assignee provided, or no information about that assignee listed). Only 2,168,220 USPTO patents have assignee address information of any kind in the database (and ∼2M have address at the resolution of city or better). As our algorithm depends strongly on geolocation, only these ∼2M USPTO patents will have even a chance of acquiring a disambiguated assignee. Over 95% of the patents filed in each office have at least one inventor geolocation, and coverage is good in the EPO and PCT for assignees as well. Only ∼90% of the assignee addresses for the USPTO result in a geolocation, due to the relatively low quality of assignee addresses

found in the data.

	EPO	PCT	USPTO
Patents in the database	2.67	2.37	4.14
Patents with inventor geolocation(s)	2.54	2.18	4.06
Patents with assignee geolocation(s)	2.58	2.24	1.95

Table 1: Patents in the database (numbers in millions of patents), and the address information included in them. Here, 'geolocations' refer to address information uploaded to YQL that returned at least one non-empty response. Empty responses are likely due to missing addresses (particularly due to the USPTO, where many assignees addresses are not provided).

2 Assignee name matching

Assignee disambiguation can be extremely difficult due to the large number of alternate spellings of assignee names. An example of a difficult disambiguation is shown in Table 2 for alternate naming for the National Institutes of Health (NIH) in the Rockville / Bethesda areas of Maryland, USA, which is an agency of the Dept. of Health and Human services. Patents produced by the NIH may have assignee names that solely include references to the Department to which it reports, or completely exclude the Department, or mention the Department in conjunction with the Institutes. While this extreme variability makes disambiguation difficult, we note that each of the addresses in this table are geolocated to the same lat/long with high quality using YQL. The geolocation is thus providing two services in this respect: in addition to locating the specific place where the institution is located, it is providing a robust disambiguation of the addresses. The similarity between the names in Table 2 in conjunction with their precise geolocation to *identical* points certainly suggests that these names likely refer to the same entity, and in general one has good reason to believe that 'similar names' found at identical high precision street address are likely to refer to the same institution. Our algorithm for the first step of disambiguation is thus to block all names according to their high-resolution geolocations and search for 'similar names.' Names that are 'similar' are linked to one another as referring to the same disambiguated institutions.

In order to determine if two names are similar, it is useful to build a list of 'common' and 'rare' words. A dictionary of 'common' words (e.g. "hospital" and "institute") that was generated by hand (and passed through google translate in a few languages) is read into memory, as well as a dictionary of location names (e.g. "Boston") provided by GeoNames and all first and last names occurring in the inventors found in our databases. All words on any of these lists are treated as 'common' in the disambiguation algorithm. A few words

patent	assignee name	assignee address
EP1807440	Department of Health and Human Services	6011 **Excecutive** Boulevard, Rockville MD 20852
EP2019710	National **Institute** of Health	Office of Technology Transfer 6011 Executive Boulevard, Suite 325, Rockville MD 20852-3804
EP1361886	The Gov. of USA, as represented by the Secretary, **Dept.** of Health and Human services, National Institutes of Health	Office of Technology Transfer, 6011 Executive Boulevard, Suite 325, Rockville, MD 20852

Table 2: Examples of three patent assignees that are "similar" but difficult to disambiguate. A few misspellings or abbreviations are bold-faced. Each of them are geolocated to at least one identical high-resolution latitude/longitudes (39.048843, −77.120419 with a quality rating of 87, specifically). There is significant overlap between the words found in the assignee name in EP1361886 and the words in the assignees of either EP2019710 and EP1807440, suggesting these names should be matched.

in the common list (e.g. "Company") were manually selected as completely uninformative, where the inclusion of these words caused many spurious incorrect matchings, and are removed from the names before matching. For the manually curated common words, a misspelling dictionary is also constructed by (a) producing all deletion errors possible by deleting each character in turn, and (b) by producing every permutation error possible by swapping the order of every character in the name. These permutations and deletions are *not* applied to the common words. In order to check to see if a name is 'common' within one error, we check to see if it is found in the common word list, or if it *or any single deletion substring* are found in the misspelling dictionary. During assignee disambiguation, words that are on the common list or within one deletion from the misspelling dictionary are treated as 'common,' otherwise they are treated as 'rare.' All of the assignee names are processed as described in Sec. 7.

To perform the first step of disambiguation, we search for similar names at each high-resolution geolocation. In this paper, "high-resolution" refers to any geolocation with a YQL quality of at least 70, generally corresponding to a geolocation with street-line level accuracy or above. Geolocations with an accuracy on the level of zip-codes, cities, or higher are ignored in this first step of the disambiguation process. The algorithm then iterates over each high-resolution lat/long, and all pairs of names at that location are compared to see whether they are similar:

1. If any two 'rare' words are within one edit distance of one another in both names, the names are a mach. If all rare words in the first name are more than one edit distance from all words in the second name, proceed to step 2. For example, "Harvard University" and "Harvard College" are matched in this step because "harvard" is a

rare word.

2. If the list of ‘common’ words in both names have at least two elements in common, the names are a match (if one of the names consists of exactly one word [e.g. “Apple”] and that word is ‘common’, only one shared ‘common’ word is required). If there is not a sufficient overlap between ‘common’ words found in the names, proceed to step 3. For example, “The General Hospital Corporation” and “Massachusetts General Hospital” are matched in this step, because ‘general’ and ‘hospital’ are common words.

3. Check to see if the first name contains an acronym found in the second name. For each word in the first name, break it into individual letters and see if there is a subset of words in the second name (preserving the ordering) that all begin with those letters. If no match is found, check for acronyms in the second name. If no acronym is found at this step, we assume the names are distinct. For example, “The Massachusetts Institute of Technology” and “MIT” are matched in this step, since ‘m’, ‘i’, and ‘t’ are found sequentially in the first letters of words in the first name.

Having linked all of the names at each lat/long, every name/geolocation pair is assigned a unique identifier, with any names matched in the algorithm assigned the same identifier. Beginning with the ~351K name/high-resolution geolocation pairs found in the raw data after geolocation and name cleaning, there are ~331K unique identifiers produced using this algorithm. We note that this is a rather modest reduction in the number of name/geolocation pairs, and may appear to have done very little. However, this step performs the essential service of cleaning very noisy names, of particular importance for large institutions. These are the clustered names that are passed to the neighborhood search described in Sec. 4.

3 Inventor name matching

Inventor names generally have less variability in their structure than assignee names, where usually there is a ‘last name’ (typically the first word in the name), a ‘first name’ (typically the second word in the name) and finally various ‘middle names.’ In reality, the first and middle names are sometimes interchangeable, a two-word last name may be separated, and additional titles or company names may be added to the name (see Table 3 for two examples). In order to overcome these types of errors, we adapt our word-based matching of names at the same high-resolution geolocation that was used to disambiguate assignees. A manual inspection shows that these errors tend to be far more common in the EPO and PCT than in the USPTO (although it is difficult to quantify the rate of any particular error type without an accurate disambiguation in hand), but the USPTO tends to have far lower accuracy in the geolocations as well.

patent	inventor name	inventor address
EP2340782	GOMES DA CUNHA PONCIANO, José Antônio	Av. Ipiranga 55 Centro,CEP: 25685-250 Petrópolis, RJ
EP2386338	GOMES, José Antonio, da Cunha, Ponciano	Av. Ipiranga 55, Centro, Petrópolis - RJ, Cep: 25685-250
EP1247533	Howard, Jr, Harry Ralph	Pfizer Golbal Res. and Dev., Eastern Point Road, Groton, Connecticut 06340
EP1220831	HOWARD, Harry Ralph, Jr. Pfizer Global Reasearch	and Development Eastern Point Road Groton, CT 06340

Table 3: Example of inventor names with a variety of errors. The first two names refer to the same person, but the person's last name is split in the second occurrence (note also the missing accent in Antonio). In the second example, 'Jr' is put in the position of the first name in one instance and a portion of the address field is added to the inventor's name in another instance. Both of these are geolocated to the same position, and a flexible matching can be performed.

In order to disambiguate the inventor names, each name at a high-resolution geolocation is processed as described in Sec. 7. A search is performed for the strings "c/o" and "c/-"; any words following this substring are forbidden from being matched at that geolocation under the assumption it refers to the assignee (matching due to that string is not forbidden at other geolocations). In each name, it is assumed that the first two words in the name correspond to the last and first names, respectively, and we check to see if these assumed first and last names are found *anywhere* in the name we are comparing.

1. Check to see if the 'last name' is found in the name we are comparing to. If the word has more than three characters, allow for a difference of one edit. We keep track of how often the 'last name' was a match to the first word in the compared name.

2. If the 'last name' was found in step 1, check to see if the 'first name' is found in the compared name. If there was *perfect* agreement in the last name, permit the 'first name' match to differ by one edit distance. If there was *not perfect* agreement in the 'last name,' require perfect agreement with the 'first name' (max. edit distance of 0).

For each pair of names, we perform this check using the first two words of each in our search. If we find a 'last name' + 'first name' match in between either of the names being compared at the same high-res geolocation, we link the names as referring to the same individual.

This algorithm is fairly robust and able to disambiguate the names of a majority of high res geolocations without difficulty. However, significant errors can occur due to the confluence of two events in the data: (1) a large number of inventors using the same address

and (2) culturally common 'middle names.' For example, the address "Prof. Holstlaan 6,NL-5656 AA Eindhoven" is used as an inventor address for over 34k EPO patents, and at that precise address there are 743 unique inventor names that have a middle name "Maria." In such cases, last names that are one edit distance away from common last names will cause an overwhelming number of incorrect links between names (e.g. there is a person named "Marra, Johannes" at the same address, with a last name one edit distance from "Maria"). To prevent these huge errors, we perform a pruning step, and unlink pairs of names if the 'last name' that caused the link was matched to a non-'last name' more than twice as often as it was matched to a 'last name.' This final post-processing step removes linked names when the 'last name' is overwhelmingly matched to a 'first' or 'middle name', indicating a spurious match.

As was the case in the disambiguation of assignee names, in our final step we assign each name/geolocation pair a unique ID, ensuring that each matched name has the same ID. From the 2,241,414 name/high-res geolocations found in the data, we produce 1,997,388 unique IDs after this round of disambiguation. These identifiers are passed to the neighborhood search described in Sec. 4.

4 Nearby exact name matches

The disambiguations in Secs. 2 and 3 provide a robust matching between similar names, but do so only at precise, high resolution geolocations. Many addresses are low resolution, where e.g. the address "Boston MA" is very imprecise, and would not be used in the name disambiguation described in Secs. 2 and 3. Any typographical errors in the address field that could also move precise addresses by even a few meters would prevent the linking of names as well (e.g. "123 Main St." vs "132 Main St."). In order to expand the coverage of the disambiguation to imprecise addresses, we perform a search for nearby geolocations (within 20km of one another, an upper bound on the typical commuting distance in major metropolitan areas in the US [4]) that have an exact name match (after cleaning capitalization and punctuation variations as described in Sec7). This provides links between high resolution geolocations where identical names are found nearby, links between high and low resolution geolocations of the same name, and low resolution IDs of a single exact name match. This is some sense the analog of the disambiguation described in Secs. 2 and 3, which used exact locations to search for similar names; in this step, we use exact names to search for nearby locations.

The names and geolocations (both high quality and low quality) for inventors and assignees are read into memory and processed as described in Sec. 7. For each unique name, only the highest quality geolocations are kept (e.g. for a name with two geolocations found using YQL, a location of quality 60 and another of quality 39, only the first would be kept). To disambiguate the names:

1. For each name occurring on any patent where YQL provided more than one geolo-

cation of the same quality, we link all of those name/location pairs into the same identity. This corrects any noise in the geolocation on an inventor's or assignee's address on a patent.

2. For each exact name, we determine the distance between all pairs of geolocations. If that distance is less than 20km, link those name/locations.

3. We assign all linked names to a unique identifier, and any alternate spellings of those names that were disambiguated following the steps in Secs. 2 and 3 are *also* assigned to the same unique identifier.

4. Finally, we assign any unlinked names their own low-resolution unique identifier.

Many names are never found in conjunction with a high-precision geolocation, and misspellings or alternate spellings in these names will not be corrected using this algorithm. For example, the patents US6495146 and US6028086 have assignee names "Pfizer Incorporated" and "P Pfizer Inc" respectively, both with the address "New York, NY." These names will *not* be linked using this algorithm, due to the simultaneous imprecise naming *and* imprecise addresses. Despite this limitation, we still have good coverage both in the number of disambiguated names and the number of disambiguated individuals and the number of patents they cover, as shown in Table 2 of the main text.

Linking all identical names at a low-resolution geolocation implicitly assumes that any identical name in the same region (city or zip code) must refer to the same individual. While this is likely true in many cases, there are also many situations where it may not be true for inventors. For example, two inventors in the vicinity of Warwick, England, are named "Jones, Christopher B" and "Jones, Christopher J." Patents invented by "Jones, Christopher" in this region *cannot* be assumed to refer to the same person, as each patent could potentially be invented by "Christopher B" or "Christopher J". We therefore search for exact name matches for in prefixes of longer names (e.g. "Jones, Christopher" and "Jones, Christopher B"), to identify potential matches due to an excluded middle name as well as potential errors due to linkages in ambiguous cases. To check for prefix matching for both assignees and inventors, we:

1. For each name n in the patent data, we search for exact prefix matches of other names $\{m_i\}$, after removing punctuation and cleaning spacing. In the case of assignee names, we remove non-informative words such as "corp" and "gmbh" in this step.

2. All pairs of IDs that are associated with the name n and one of the m_i's are treated as potentially linked. IDs that are potentially linked to *exactly one* other ID by prefix matching are merged into a single ID.

3. Steps 1-2 are repeated until no new merges occur.

4. After Step 3 is completed, any inventor IDs that are potentially linked to *more than one* other ID are treated as erroneous. These IDs are split apart, with every patent held by the original ID being given its own unique ID. This step is not performed for assignees, only inventors.

The iterative process of linking names with single prefix matches assumes that inventor names may sometimes drop the middle name. The splitting ensures that if two middle names are encountered nearby for the same first and last name, we relax that assumption. The prefix search is also performed for assignees (e.g. "Sony" and "Sony Electronics" would be linked if there was only a single match nearby), but unlike inventor IDs we do not break apart the assignee IDs associated with shorter names if more than one match is seen. This is due to the significant variability in the names of assignees: an ID with the name "Sony" may be close to other IDs with names "Sony Electronics," "Sony Entertainment," "Sony Games," etc. While we do not merge assignee IDs if more than one prefix match is found, it would be unreasonable to break apart the ID associated with "Sony" due to this variability. Testing shows that the quality of the assignee disambiguation is significantly reduced by splitting assignees in this manner, but that splitting inventors does improve all quality indicators.

5 Linking patents without geolocations to the disambiguation

The disambiguation methods described in Secs. 2-4 cannot be applied to inventors or assignees that are geolocated with very poor quality or that did not provide an address. Our geolocation of all addresses was performed on the January 2014 data provided by the OECD, but we have applied this algorithm to the more recent July 2014 OECD database for the EPO and PCT. New addresses would not be included in the geolocation process, and would be treated as being unlocated as well. Incomplete geolocations are generally due to poor quality assignee addresses in the USPTO (see Table 5).

(counts in millions)	EPO	PCT	USPTO
Patents with at least one unlocated assignee	0.11	0.14	1.66
Patents with at least one unlocated inventors	0.29	0.28	0.33

Table 4: Number of incompletely geolocated patents in each office, counts in millions. The overwhelming majority of incomplete geolocations are due to assignees in the USPTO, where address information is often on the city level at best and completely blank at worst.

Each patent having an unlocated name (either inventor or assignee) may still have some inventor geolocations, as some inventors may have provided a high quality address. In order to link these unlocated names to the previous steps of the disambiguation method, we first search for exact name matches between unlocated assignees or inventors, having name n and previously disambiguated assignee or inventor names, and we use the inventor geolocations as a proxy for the unknown name's location:

1. For each unlocated name n on patent p, having a list of $\{x_i\}$ previously geolocated inventors, search for identical names n in the previous disambiguation (after removing capitalization, spelling errors, and non-informative words as in Sec. 7). Create a potential link from the patent to any ID $\{y_i\}$ having that name n.

2. If any geolocated inventor x_i on this patent is within 20km of a matched ID $\{y_i\}$, link that patent to the nearby ID. If more than one ID is within 20km, link the patent to the one with the most disambiguated patents.

This process links names based on physical proximity, but does not permit any spelling errors or name variations due to the requirement of identical names.

In order to account for noise in assignee (or inventor) names, we also search for disambiguated inventors on patent p that are also inventors on patents with assignees (or inventors) of a similar name. Similarity for inventors in this process is more restrictive than it was in Sec. 3: we require all words in the shorter name to be found *or* an abbreviation to be found (in both cases words can be in any order, up to one spelling error total in words of length ≥ 4). Assignee similarity is the same as in Sec. 2. These shared inventorships are used to draw links to the previous disambiguation:

1. Construct a list of patents p that are missing an assignee (or inventor) location and all disambiguated inventor IDs $\{x_i(p)\}$.

2. For each x_i, construct a list of disambiguated co-assignees (or co-inventors), $\{y_j\}_{x_i(p)}$.

3. For each patent missing a location on name n, search for the most commonly occurring ID y_j that is similar to the unlocated name n (using the methods of Sec. 2 for assignees and the more restrictive name matching for inventors).

This uses our disambiguation, rather than inventor geolocations, to attach an unlocated name on a patent to a disambiguated ID. This step permits some spelling correction of names when linking the patents to the disambiguation, but as there is no geolocation information on the linked patents, we do not treat the linked IDs as 'high-quality' in our analysis.

Note that this second procedure can introduce noise in the number of raw names that are associated with an ID. For example, "The United States of America as represented by the Department of Health" and "The United States of America as represented by

the Department of Veterans Affairs" are by all respects similar names: the edit distance between them is 80% of the shortest length, and the number of identical words is over 80% of the total number of words in them. An inventor that predominantly works with the Dept. of Health and Human Services (HHS) but who collaborates with the Dept. of Veterans Affairs may cause these two entities to be linked incorrectly. For the HHS, we find these sorts of errors account for well under 5% of the total number of patents assigned to its disambiguated ID, so these errors are relatively rare.

6 Mobile inventor disambiguation

The approaches to disambiguation in Secs 2-5 provide a disambiguation of institutions and inventors, but only within a ∼20km radius. While assignees are generally expected to remain in a fixed and localized position (neglecting the possibility that the same company moves from one city to another), inventors are far more likely to move between cities or countries. Our algorithm would be incapable of linking the names of mobile inventors, since the distance between their geolocations would be well above our threshold of 20km. In order to overcome this limitation, we must add one final step to the disambiguation algorithm that allows for inventor name matching over greater distances. We do this by searching for similar names that have other important characteristics in common, suggesting that they are the same person. In particular, we link a pair of disambiguated inventor IDs that share an *exact* name match if any of the patents held by those two inventors

1. share a disambiguated co-inventor
2. share a disambiguated co-assignee
3. are members of the same triadic family
4. have at least one citation from one to the other

Note that shared technological classes are not included in our measure of similarity, due to the varying levels of aggregation available (raging from a handful of WIPO industrial sectors to tens of thousands of 7-digit IPC codes) and the unknown technological and cognitive distance between classes.

Inventor names that are similar (requiring all words in the shorter name to be found in the longer name) are taken to be the same individual if they have *two* of the characteristics in common. While the primary purpose of this step is to detect the long-range mobility of inventors between regions or countries, we note that this permits spelling error correction in inventor names within the 20km range that were not possible in step 2 of the algorithm. IDs linked in this step are thus labeled as 'high quality,' as they incorporate some level of geolocation and spelling correction.

7 String handling

When parsing the names, all latin characters are converted into lowercase letters. All accented letters are converted into the character ‘x’ (this has the affect of penalizing the removal of an accent, but not penalizing the change of an accent in a name). All symbols (e.g. ‘-’ or ‘.’) are converted into spaces. After this, words composed of a single character are deleted and all double-spaces, triple-spaces, etc. are converted to a space.

For assignees, a specific list of ~ 60 words are deleted from the names before passing through to disambiguation (for example, “inc”, “corporation”, “aktiengesellschaft”, and “the”). The designation of words to completely delete before disambiguation were made manually, and altering this list may change the results to some extent.

For inventor names, a few words are dropped from the names: “dr”, “de”, “da”, “di” “mc”, “von”, “der”, “van”, and “den”. These short words tend to be non-informative within the last name of individuals, and cause problems with our algorithm if they are treated as a ‘first name.’

References

[1] OECD REGPAT database (January 2014).

[2] Li, G. *et al.* Disambiguation and co-authorship networks of the us patent inventor database (1975–2010). *Research Policy* **43**, 941 (2014).

[3] OECD REGPAT database (July 2014).

[4] Kneebone, E. & Holmes, N. The growing distance between people and jobs in metropolitan America (2015).
